# Sodium Alginate—Natural Microencapsulation Material of Polymeric Microparticles

**DOI:** 10.3390/ijms232012108

**Published:** 2022-10-11

**Authors:** Olimpia Daniela Frent, Laura Gratiela Vicas, Narcis Duteanu, Claudia Mona Morgovan, Tunde Jurca, Annamaria Pallag, Mariana Eugenia Muresan, Sanda Monica Filip, Roxana-Liana Lucaciu, Eleonora Marian

**Affiliations:** 1 Department of Pharmacy, Faculty of Medicine and Pharmacy, University of Oradea, 29 Jiga Street, 410028 Oradea, Romania; 2 Doctoral School of Biomedical Science, University of Oradea, 1 University Street, 410087 Oradea, Romania; 3 Faculty of Industrial Chemistry and Environmental Engineering, Politehnica University of Timisoara, 2 Square Victoriei, 300006 Timisoara, Romania; 4 Department of Chemistry, Faculty of Informatics and Sciences, University of Oradea, 1 University Street, 410087 Oradea, Romania; 5 Department of Preclinical Discipline, Faculty of Medicine and Pharmacy, 1st December Square 10, University of Oradea, 410087 Oradea, Romania; 6 Department of Physics, Faculty of Informatics and Sciences, University of Oradea, 1 University Street, 410087 Oradea, Romania; 7 Department of Pharmaceutical Biochemistry and Clinical Laboratory, Faculty of Pharmacy, “Iuliu Hatieganu” University of Medicine and Pharmacy, 8 Victor Babes Street, 400012 Cluj-Napoca, Romania

**Keywords:** sodium alginate, microencapsulation, microparticles, natural polysaccharide

## Abstract

From the multitude of materials currently available on the market that can be used in the development of microparticles, sodium alginate has become one of the most studied natural anionic polymers that can be included in controlled-release pharmaceutical systems alongside other polymers due to its low cost, low toxicity, biocompatibility, biodegradability and gelatinous die-forming capacity in the presence of Ca^2+^ ions. In this review, we have shown that through coacervation, the particulate systems for the dispensing of drugs consisting of natural polymers are nontoxic, allowing the repeated administration of medicinal substances and the protection of better the medicinal substances from degradation, which can increase the capture capacity of the drug and extend its release from the pharmaceutical form.

## 1. Introduction

Sodium alginate is a natural polysaccharide with a linear structure, is biodegradable, biocompatible and safe for the body, provides strength and flexibility to the tissue, and can be used industrially because it has gelling, viscous and stabilizing properties and the ability to retain water. Alginate can be synthesized from the cell wall of various species of brown algae: *Laminaria hyperborea*, *Ecklonia maxima*, *Ascophyllum nodosum*, *Eisenia bicyclis* and *Macrocystis pyrifera* ecc., and from various species of bacteria: *Azotobacter* and *Pseudomonas*. From these sources, alginate extracted from brown algae has commercial importance for the food, pharmaceutical, cosmetic industries, etc. [1,2,3,4,5].

The extraction of alginates from brown algae is carried out in alkaline medium with sodium carbonate, sodium hydroxide or aluminum hydroxide, in several stages after the collected algae have been dried and shredded. The extract obtained is subjected to precipitation with sodium chloride or calcium and to the filtration operation, the precipitate formed (sodium/calcium alginate) is converted into alginic acid by treatment with diluted clorhydric acid, and the alginic acid is converted into a dry sodium alginate powder. The alginate obtained in order to be used must undergo chemical treatments to remove impurities (e.g., heavy metals, endotoxins, proteins, carbohydrates and polyphenols) and then turned into powder. In order for alginate to be able to be used in the biomedical and pharmaceutical field, it must be safe for the body and biocompatible, that is, it must have high purity. A crude alginate purified by a multistage extraction method is devoid of or contains impurities in a low amount and can be taken orally without causing a response from the immune system [2,3,6,7,8,9].

Nowadays, the development of microencapsulated pharmaceutical forms has become an attractive and widely used field in pharmaceutical technology because over time, they have proven to be safe and effective drug release systems. The emergence of new processible, biocompatible, biodegradable and nontoxic biomaterials for the body in the field of medicine have made it possible to develop much more efficient and much more advantageous pharmaceutical systems than classical pharmaceutical forms. Thus, the category of new high-performance materials includes anionic natural polymers, such as sodium alginate, that are considered advantageous microencapsulation materials and which according to studies can influence the kinetics of the release of the drug from the matrix, according to their degradation in the body [10,11]. When developing particulated pharmaceutical forms, some of the main objectives of the formulation are to maintain the rate of release of the medicinal substance at an effective therapeutic level, with controlled speed and release time, and to protect the medicinal substances from gastrointestinal, enzymatic degradation, etc., and from the action of external factors [12]. In this review, a short presentation will be made about the chemical structure, properties and possibility of using sodium alginate as an anionic polymer when developing microencapsulated pharmaceutical forms of the microsphere type.

## 2. Chemical Structure of Alginate

According to the information of Phillips G.O. and Williams P.A. and of Lee K.Y. and Mooney D.J., until 1958, information about the chemical structure of alginate suggested that sodium alginate is predominantly made up only of β-D-manuronic fractions, but it was later observed that α-L-glucuronic acid fractions are also present in its structure. The ratio in which the two fractions are present in its structure varies according to the natural source from which it was extracted [3,9,13]. 

Sodium alginate is considered to be a polyanionic copolymer which structurally is the sodium salt of alginic acid, an acid consisting of several successive groups of the two uronic acids: β-D-manuronic acids (M) and α-L-glucuronic (G), linearly linked to each other by 1–4 glycosidic bonds [2,4,5,14,15,16].

**Figure 1:**
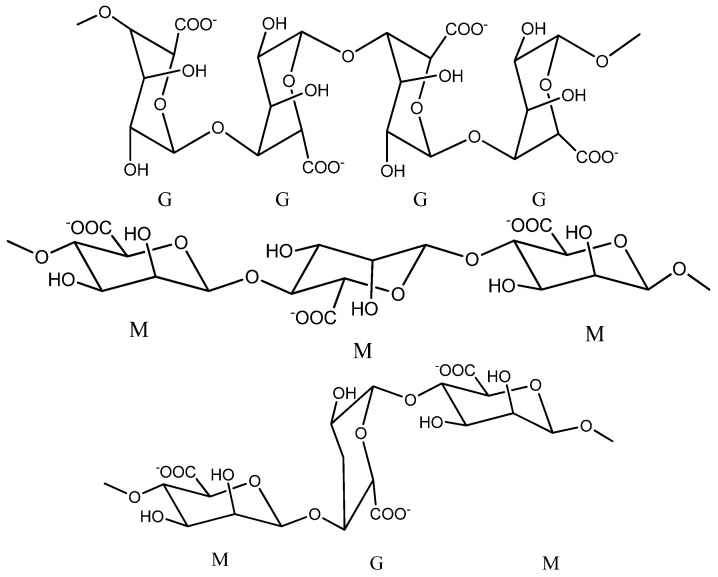
Chemical structure of sodium alginate (Chemdraw scheme).

It has the chemical formula (C_6_H_7_NaO_6_)_n_ and an average molecular weight of 216.121 g/mol [17]. By a partial hydrolysis reaction in acidic medium, the alginate molecule can be cleaved into three successive fractions: manuronics (MMMMM), glucuronics (GGGGG) and a mixture of manuronic fractions with glucuronics (MGMGMG), as shown in Figure 1 [9,18].

Studies show that the proportion of the two fractions M and G and the length of the chains in the alginate structure may vary from one species of brown algae to another, so the alginate that is extracted from *Laminaria digitata* and *Ascophyllum nodosums* presented a ratio of 1.16:1.82 between the two fractions M and G [19]. Glucuronic chains give alginate many advantages, such as their possibility to participate in cross-linking with calcium ions and the possibility of forming gels with superior mechanical properties [9].

## 3. Physico-Chemical Properties of Sodium Alginate

From the physico-chemical point of view, sodium alginate is presented in the form of a solid powder that is white or slightly yellowish and hydrophilic, dissolves easily in water and has the ability to form gels in the presence of divalent ions, all of which make alginate a useful material for the delivery of medicines and cellular immobilization [5,6].

The physico-chemical properties of alginates (mechanical properties, swelling and diffusion capacity) are influenced by several characteristics: the composition and arrangement of the two groups of uronic acid in the structure, the molecular weight of the polymer, the type of functional groups in the structure and the concentration of the reticular agent used [2,7,20]. The characteristics of alginates may vary depending on the natural source from which it was extracted and the season and the geographical location from which the plant was harvested [6].

### 3.1. Physico-Chemical Properties

#### 3.1.1. Molecular Weight

Commercial sodium alginate has a high molecular weight between 32,000 and 400,000. It has long M and G chains in the structure and a polydispersion index that varies between 1.5 and 3 (Mw/Mn). Studies show that the viscosity of alginate solutions is influenced by the molecular mass and pH of the reaction mass, so the viscosity increases with a decrease in pH and reaches a maximum around pH 3–3.5, because at this value, the carboxyl groups in its structure become protonated and can form hydrogen bonds. Increasing the molecular weight of alginate increases the rate of gelling and the physical properties of gels (tensile strength, elasticity, viscosity).

However, sometimes too much of an increase in molecular weight can lead to a very viscous solution of alginate, which is undesirable in certain situations [9,21,22]. For example, in the preparation of alginate hydrogels used as a cell immobilization matrix (in the case of vaccines), if the alginate solutions used are too viscous, the viability of the cells during the hydrogel formation process may be reduced by the high shear forces applied when mixing them with alginate. Cell membranes in general are highly sensitive to mixing, and sometimes strong mixing can cause cell death [23].

#### 3.1.2. Solubility

The solubility of sodium alginate in cold water is slower and leads to obtaining a viscous solution. It is insoluble in alcohol, hydroalcoholic solutions with alcohol content above 30%, chloroform and ether [4]. Studies show that its solubility depends on the pH, molecular weight, ionic strength, nature of the ions present in the structure and concentration [24]. The pKa value of guluronic acid is 3.6, while that of manuronic acid is 3.3. Compared to sodium alginate, calcium alginate is insoluble in water and organic solvents, but is soluble in sodium citrate [22].

#### 3.1.3. Stability

Sodium alginate is compatible with most anionic substances and with few cationic substances, and it shows higher stability against external factors if it is conditioned in the form of a dry powder than in the form of a solution. With acids, sodium alginate gradually forms a gel of alginic acid at low pH values; at elevated pH values, alginic acid dissolves and restores its original viscosity. In alkaline environment, sodium alginate can withstand short periods of time, since pH values higher than 11 reduce its viscosity. In the short-term, sodium alginate can withstand high temperatures, so it can be sterilized, but in the long term, the high temperature in sterilization can reduce the degree of viscosity [25].

### 3.2. Mechanical Properties

#### 3.2.1. Viscosity

The viscosifying capacity of alginate is dependent on the molecular weight and concentration of the polymer, and gelling (affinity for cations) depends on the amount of glucuronic acid in the structure. Thus, in the structure, the higher the amount of glucuronic acid that is found, the more the solubility of alginate in water and the gelling capacity increases, resulting in a more resistant, viscous, strong and more stable gel [2,5,25,26]. According to studies, sodium alginate solutions are not Newtonian fluids but pseudoplastic fluids whose viscosity changes drastically when they are dissolved in water and diluted with water [4].

Studies show that the viscosity of alginate is dependent of temperature. The thermal and viscoelastic properties of alginate films can be studied using differential scanning calorimetry (DSC). DSC studies on various thermosensitive alginate gels obtained in the temperature range between 0 and 100 °C showed low rigidity at high temperature. It appears that at temperatures below 100 °C, the noncovalent bond between the adjacent polymeric groups kept the alginate intact under oscillatory conditions of deformation, but this equilibrium was interrupted by a constant magnetic stirring [2].

Commercially used sodium alginate has varying degrees of viscosity, and the resulting 1% aqueous solutions have viscosities that can vary from 20 to 400 cP (centipoise) and 0.02–0.4 PaS (pascals per second) at 20 °C [22].

#### 3.2.2. Mucoadhesion

Alginate has good mucoadhesive properties due to the presence of free carboxyl and hydroxyl groups in the structure. In the physiological environment, electrostatic repulsive forces occur between alginate and mucin due to negative charges of sialic acid, sulfate groups in the mucus structure and anionic carboxylic groups of alginates. This suggests that the bioadhesion between mucin and alginate is achieved through intra- and intermolecular hydrogen bonds. Studies claim that the mechanism of mucoadhesion follows several stages: the first stage consists of intimate contact with the mucosa when wetting and swelling of the polymer occurs, and the last stage consisting in the formation of hydrogen bonds through the processes of interpenetration of the mucin with the polymer chains [6]. This property is an advantage in administration of medication to mucous membranes because it increases the contact time and adhesion of the drug to the site of action and also increases the bioavailability of medicines [4].

### 3.3. Biological Properties

The FDA (Federal Drug Administration) has approved the use of sodium alginate in the food, biomedical and pharmaceutical fields due to its biological properties, i.e., lack of toxicity and immunogenicity, biocompatibility and biodegradability [24].

#### Biocompatibility, Toxicity, Immunogenicity and Biodegradation

Studies shows that sodium alginate can be included as an excipient in various pharmaceutical forms intended for oral administration because it is safe, nontoxic and does not accumulate in the body. Due to its chelating capacity, it can bind to various heavy metals present in the intestine protecting the body from their effects. However, when it is intended to be used in implantology or intravenous administration, the factors that can influence its biocompatibility and immunogenicity should be taken into account, such as the chemical composition (ratio of G/M groups), purification process, nature, quantity and impact of residual contaminants. Many studies claim that the use of commercial alginate by parenteral route can cause fibrosis and immune response. In order for alginate to be safe for the body and to be used in the biomedical field, it must be prepared and purified very carefully by decontamination methods during the extraction process in order to remove all traces of heavy metals, endotoxins, proteins and phenolic compounds with immunogenic potency [24,27]. The enzymatic degradation of alginate in mammals is not possible due to the absence of alginase, an enzyme involved in the process of undoing the polymer chains, and medium- or high-molecular weight alginates cannot be eliminated renally entirely because they are filtered more slowly by the kidneys. Taking into account the problem of biodegradation, studies show that alginate can be degraded by oxidative way, ionic reticular, etc., if it is subjected to structural changes [24].

### 3.4. Other Properties

#### 3.4.1. Ionic Reticular Capacity of Alginate with Ca^2+^ Ions

**Figure 2:**
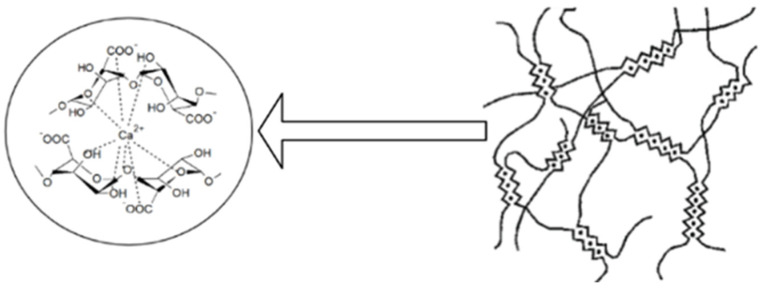
Formation of the three-dimensional network of the “egg box” type by the reticulating sodium alginate with Ca^2+^ ions (scheme made with the Biorender program).

Alginate can form, by ionic reticulation with polyvalent cations, three-dimensional gels which have a rigid, orderly and strong structure. Agulhon P. et al., showed that the reticulation that is made between alginate and alkaline-earth cations is of an electrostatic nature, and that between alginate and the cations of transitional metals, it is covalent. This is due to the interaction of free carboxyl or hydroxyl groups of the G fractions in the alginate structure with bivalent/polyvalent cations under controlled temperature conditions [2,6,7,9,28]. The affinity of polyvalent ions to alginate is different following the order: trivalent cations > Pb^2+^ > Cu^2+^ > Cd^2+^ > Ba^2+^ > Sr^2+^ > Ca^2+^. Studies show that of the bivalent ions, Ba^2+^ and Sr^2+^ can form stronger micro-/nanoparticles of alginate than Ca^2+^ ions, although Ca^2+^ ions are the most used even if they do not have the highest interaction power. Ca^2+^ ions are the most preferred for the development of microparticles because they are the safest for the body, and through reticulation, they form an adequate network of gel of Ca-alginate in mild conditions [6,12]. The use of Pb^2+^, Cu^2+^ and Cd^2+^ is limited due to their toxicity [6]. In the literature, gelling is presented as an “egg box” type of network that is formed when Ca^2+^ ions replace the Na^+^ ions in the alginate structure, binds crosswise and is antiparallel to two alginate molecules [29], as shown in Figure 2.

However, the binding of alginate with calcium ions can be influenced by temperature in the sense that at low temperatures, the reticular capacity of alginate decreases. A slower reticulation leads to obtaining ordered gelatinous networks with improved mechanical properties. The mechanical properties of ionic reticulate alginate gels may also vary depending on its chemical structure: for example, gels obtained from alginate with a high content of G fractions are more rigid than those containing a small amount of M fractions [9]. Studies have shown that microencapsulated pharmaceutical forms consisting only of alginate and Ca^2+^ have some shortcomings compared to those consisting of two polymers and Ca^2+^ ions: the gelling process is formed instantly and cannot be controlled due to the increased solubility of alginate in water, the gels they form are not stable in the long term under physiological conditions, the polymeric matrix that is obtained is easily degraded in acidic medium, and it is porous and permeable, from which large amounts of the drug can be lost during preparation, making it difficult to control the release of the drug. Microparticles obtained by ionic reticulation from alginate and calcium ions are much more rigid, unlike microparticles obtained by coacervation from alginate and various natural or synthetic polymers that are much more flexible. By complexing sodium alginate with other natural polymers (e.g., chitosan) and with calcium ions, the physico-chemical properties of the preparations are considerably increased by increasing the stability of the dosage form, by limiting the loss of the medicinal substance and by improving the release profile of the active substance due to the decrease in the porosity of the pharmaceutical form [9,27,30,31,32,33,34].

Currently, reticulation of alginate has also been attempted with other natural polymers: gelatin [35], carrageenan [36], cellulose [37], pectin [38], acacia gum [39] and hyaluronic acid [40]; synthetic polymers: polyethylene glycol [41] and polyacrylamide [42,43]; proteins: ovalbumin [44]; polypeptides: poly L-glutamic acid [45], etc., to enhance its gelling properties and improve the final properties of pharmaceutical forms. Thus, by reticulating alginate with pectin in the presence of Ca^2+^ and by plasticizing with 10% glycerol, it is possible to obtain polymer films with low solubility in water that are flexible and have adequate swelling capacity [46]. Collagen reticulation proved to be advantageous because it managed to maintain the neural cells viable throughout the encapsulation process in the 3D network of the hydrogel [47]. Pires A.R.L. et al., managed, through an advantageous reticulation of alginate with chitosan and polydimethylsiloxane, to produce a bandage with wound-healing capacity, which was observed by thrombogenicity and hemolysis tests [48]. Babu V.R. et al., by reticulation of alginate with methylcellulose and glutaraldehyde, synthesized effective microspheres with controlled release of nifedipine [49].

#### 3.4.2. Complex Coacervation Capacity of Alginate with Chitosan

Coacervation is a process of physicochemical microencapsulation [50] in which two different colloidal phases, one rich in polymeric particles, called the “coacervate phase”, and the other poor or totally devoid of polymeric particles, called the “equilibrium phase”, separate into coacervate microparticles when they come into contact with each other [51,52,53,54]. Liquid medicinal substances (in the form of emulsion), solids (in suspension form), hydrophilic or hydrophobic medicinal substances and living cells may be encapsulated in microparticles by the coacervation technology, provided that the active substances are insoluble or very poorly soluble in the polymeric matrix/coating and are compatible with the polymer used in microencapsulation [53]. According to the factor causing the desolvation, the polymeric systems involved in the reaction and the phase separation mechanisms, coacervation can be of two kinds: simple coacervation, which generally occurs in the presence of a single polymer through the dehydration mechanism caused by the addition of an electrolyte/salt/desolvating liquid to the reaction medium, or complex coacervation, which occurs in the presence of two or more incompatible polymers by an electrostatic reaction [52,53,54,55,56].

**Figure 3:**
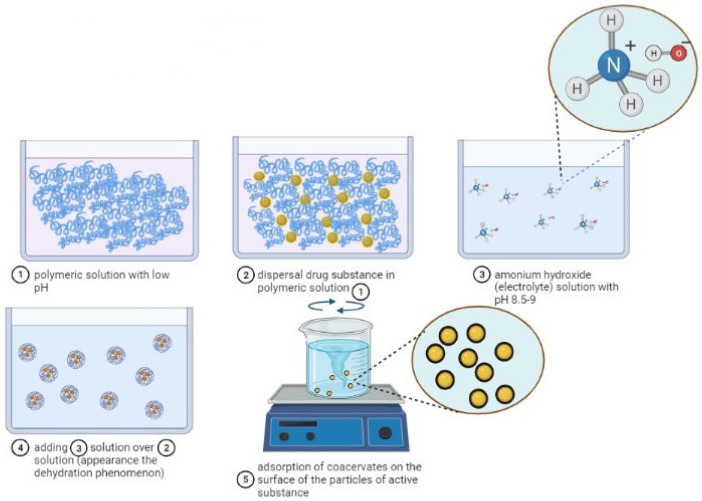
The mechanism of microparticles formation by simple coacervation (scheme made with the Biorender program).

By simple coacervation, according to Figure 3, microencapsulated polymeric pharmacokinetic systems can be synthesized as follows: particles of medicinal substances are dispersed in a low-pH polymeric solution. A solution with high pH (8.5–9.0) of ammonium hydroxide, which is strongly hydrophilic, is added to the colloidal system in order to form the baking drops which are then adsorbed to the surface of the particles of the medicinal substance. The process of forming the microspheres is carried out under stirring, with high mixing speed, in order to avoid bonding and the formation of agglomerates, and then filtration. By this method, microparticles with the size ≤10 nm [52,55,57,58,59] can be obtained.

**Figure 4:**
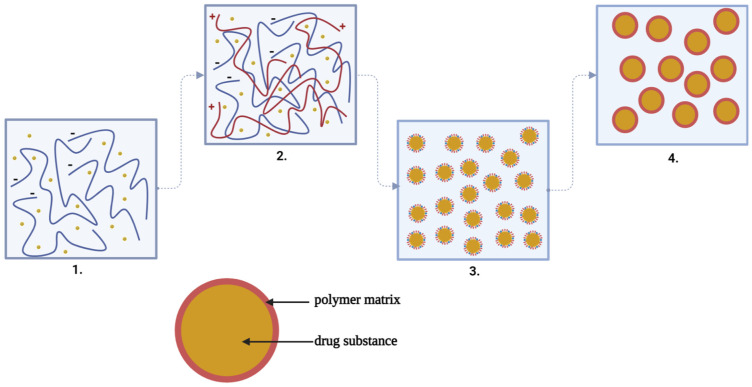
The steps of complex coacervation: 1. Suspension of the drug substance in the solution of the anionic polymer (−). 2. Adding the anionic polymer solution with drug substance to the droplets in the solution of the cationic polymer (+). 3. The adsorption of the coacervate at the surface of the particles of the active substance forming a continuous gelatinous envelope around it. 4. The polymeric matrix solidification/hardening at high temperature (drying) and the separation of the microcapsules by centrifugation or filtration takes place [53,54] (scheme made with Biorender program).

By coacervation, the microparticles are formed according to a mechanism consisting of four stages, as can be seen in Figure 4, and are based on a three-phase system represented by the solvent, the active substance and the covering material:

At the first stage, the preparation of the aqueous solutions of the two polymers and the suspension or emulsification of the active substance solid or liquid in the solution of the anionic polymer forming a hydrophobic phase takes place;

Then, coacervation takes place by adding the hydrophobic phase to the droplets in the aqueous low-polymer (cationic) environment and by the separation of the phases by electrostatic interaction of the two polymeric media, favored by the reaction medium and pH;

At the third stage, the adsorption of the coacervate takes place at the surface of the particles of the active substance, forming a continuous gelatinous envelope around it;

Finally, the polymeric matrix solidification/hardening at high temperature (drying) and the separation of the microcapsules by centrifugation or filtration takes place [53,54].

In simple coacervation, phase separation occurs due to incompatibility thermodynamics or repulsion between molecules, and in complex coacervation, the separation of phases occurs in aqueous solution due to the strong affinity of the oppositely charged species [60]. Microparticles obtained by complex coacervation compared to those obtained by simple coacervation are insoluble in water, have excellent controlled release properties and are resistant to heat [61].

The advantage of complex coacervation is that in the preparation, a wide range of natural, synthetic or semisynthetic polymeric substances can be used as microencapsular materials, such as chitosan, alginate, carboxymethylcellulose, gelatin or polyethylene glycol, etc., with different electrical charges [52,53].

Currently, complex coacervation is one of the most effective methods of microencapsulation in both the food and pharmaceutical fields [54]. In the pharmaceutical field, a wide range of pharmaceutical forms can be prepared by complex coacervation, such as microcapsules [62], microspheres [63], hydrogels [64], nanoparticles, etc., because it proved to be an advantageous method, as seen in Table 1. Biswas S. et al., encapsulated a measles antigen in nanoparticles by ionotropic gelling (mixing) of chitosan with sodium tripolyphosphate and covering the nanoparticles obtained with a layer of alginate under light stirring at room temperature. The vaccine formulation method has been shown to be advantageous, the formulation being able to protect the antigen at oral administration from enzymatic and gastric degradation [65]. Mixing with tripolyphosphate and alginate coating of chitosan microspheres also gave good results in the encapsulation of heparin in microparticles for oral administration. Thus, the microparticles showed a particle size of 335 nm, which is optimal for oral administration, and in vitro studies suggested that over 75% of the drug substance successfully crossed the intestinal epithelium [66].

**Table 1:** Micro-/nanoparticles prepared by complex coacervation.

Obtained PF	Polymers Used	DS	Advantages of the Method of Complex Coacervation	Ref.
*MPs*	Ch, CMC	Indomethacin	Modified-release PF with few adverse effects were obtained	[62]
*Ms*	Ch, Gelatin B	Tramadol	Reducing the frequency of dosages	[63]
*NPs*	Ch, Na-Alg	Insulin	The possibility of directing the manifestation of the effect to a specific target such as the colon	[67]
*Mc*	Ch, Na-Alg	Amoxicillin	Increased patient compliance	[68]
*NPs*	Ch, Na-Alg	Nifedipine	Obtaining PF with a size appropriate to absorption at GI level	[69]
*Ms*	Na-Alg, Ch	Selenium	Allows one to obtain fast-release PF in phosphate buffer solution (pH = 7.4)	[70]
*Ms*	Ch, Na-Alg	Quercetin	Allows the encapsulation in the PF of some hydrophobic DS	[11]
*Ms*	Ch, Gelatin B	Ketorolac tromethamine	The low degree of crystallinity is an advantage for controlled release	[71]
*Ms*	Na-Alg, Ch	Isoniazid	The type of polymers included in the matrix can extend the duration of release of the DS	[72]
*Ms*	Na-Alg, Gelatin B	Buryti oil	By using this encapsulation method, certain DS of polyphenolic type or volatile oils are protected from attacks of environmental factors	[73]
*Ms*	Na-Alg, Ch	Prednisolone	Rough PF can be obtained, with a similar appearance, wrinkled/smooth at the surface, with a compact structure and large number of folds, stable from temperature, and can be used at normal physiological temperature as delivery systems of the drug	[74]
*Ms*	Na-Alg, Ch	Prednisolone	Avoids the use of toxic reticular chemical agents	[75]
*Mc*	Na-Alg, Gelatin A	Astaxanthin oleoresin	Allows the obtaining of Ms with a high degree of entrapping and release of the embedded ingredients	[76]
*Mc*	Na-Alg, Ch	Triamcinolone	The use of Ch with high molecular weight together with Na-Alg has been observed to lead to Ms of lower sizes, mucoadhesive with better release rates	[77]
*Mc*	Na-Alg, Ch	Nitrofurantoin	Limitation of the occurrence of GI side effects manifested by nausea and vomiting given by certain DS (nitrofurantoin) following oral administration	[78]
*MPs*	Na-Alg, Gelatin B	Ginger volatile oil	Allows one to obtain PF with high stability to light, heat and oxygen	[79]
*MPs*	Gelatin, gum arabic	Lutein	Obtained particle have good stability at light, heat and oxygen	[80]
*Mc*	Gelatin, Na-Alg	Eugenol	If one of the polymers of the matrix is Na-Alg, it can potentiate the antioxidant effect of MPs	[81]
*Mc*	Gelatin B, corn oil, acacia BP 1993, bloom strength 225	Vit.A palmitate	Allow the incorporation of large amounts of lipophilic drugs	[82]
*Mc*	Ch, karaya gum, paraffin oil, formaldehyde	Diclofenac sodium	It favors the sustained release of the active ingredient from the particulate system	[83]
*Mc*	Na-Alg, HACC	Tea tree	Obtained PF with spherical shape and antimicrobial effect	[84]
*Nc*	Acacia, gelatin	Capsaicin	Obtained spherical and stabile particulate system	[85]
*Mps*	Ch, Na-Alg, CMC	Tanic acid	Could be used in formulations for dental abscess and superficial tissue treating wounds	[86,87]
*Mps*	I-carrageenan, Ch, gellan	Curcumin	These PF can be destined for oral administration with the colon as the therapeutic target for the controlled drug release	[88,89]
*Ms*	Na-Alg	Stellaria media	Such microspheres can be destined for oral administration.	[90]

Legend: Ch—chitosan, CMC—carboxymethylcellulose, Na-Alg—sodium alginate, MPs—microparticles, Ms—microspheres, Mc—microcapsules, NPs—nanoparticles, Nc—nanocapsules, PF—pharmaceutical forms, GI—gastrointestinal, DS—drug substance, EC—ethylcellulose, HPMC—hydroxypropylmethyl cellulose, vit.A—vitamin A, HACC—quaternary ammonium salt of chitosan.

The carboxyl groups, negatively charged from the alginate structure, can interact ionically with the positively charged amino groups that are embedded in the chitosan structure in the process of complex coacervation [14,31,34,52,91]. When alginate and chitosan interact ionically, the solubility of alginate at alkaline pH is prevented by chitosan, and the possible dissolution of chitosan at acidic pH is prevented by alginate because alginate dissolves only in an alkaline medium, being insoluble in an acidic medium, and chitosan dissolves only in an acidic medium, being insoluble in an alkaline medium. Thus, studies show that the complex coacervation of chitosan with alginate can be a good alternative for the development of microparticles loaded with unstable medicinal substances in different pH environments, to oral administration including proteins, antibiotics, etc. [18,27]. The technique of obtaining spherical, smooth and intact microparticles from chitosan and sodium alginate is based on two preparation methods: mixing the two polymers/adding to droplets and/or coating the obtained pharmaceutical form with a polymer layer [18].

Although the complexation of the two polymers is carried out instantly when mixed into aqueous solutions, many studies have reported that the formation of microspheres by complex coacervation can be affected by certain factors: ratio, molecular mass, nature of polymers, temperature, ionic power, pH of the reaction medium and charge densities [54,57,92,93,94]. The type and concentration of polymers may affect the mechanical properties of the polymeric matrix/coating of microparticles. If high-molecular-weight chitosan is used in the preparation, the microencapsulated pharmaceutical forms will have a much higher mechanical stability than those prepared from chitosan with lower molecular weight [18]. The reaction medium must have a pH around 5 in order to produce the interaction between alginate and chitosan and to form the polymeric matrix. A pH value greater than 7 prevents the interaction between the two polymers and causes the chitosan molecule to not be positively charged at this value. Studies show that at acidic pH, carboxyl groups in the structure of alginate by protonation form a layer of insoluble alginic acid at the surface of the microparticles and has the role of preventing the penetration of the external fluid inside the microparticles. Instead, the amino groups in the structure of chitosan at acidic pH are converted into soluble NH_4_^+^ groups which can interact with the protonated carboxylic groups of alginates reducing the process of swelling and cracking of the surface of the microparticles, preventing the penetration of water molecules inside the microparticles. Therefore, the reticulation of alginate with chitosan in acidic medium leads to a firmer, denser formulation, is resistant to decay and provides better protection of the microencapsulated material [18]. The stirring rate is involved in particle size control; the pH of the reaction mass can influence the degree of ionization of polymers because only at a certain pH value does coacervation occur the amount of salt, i.e., NaCI, used in the coacervation process can change the ionic power of the solution (too small or too large a quantity of salt can weaken the electrostatic interaction between polymers); calcium salts exhibit a different behavior than sodium salt; at low temperature, a higher yield of coacervation occurs due to the increased interaction between solvent and solute; and too high concentrations of polymers can alter the free movements of molecules, reducing the interactions between them [54,94].

Thus, when developing chitosan microspheres, a number of physical, chemical, technological, pharmaceutical and biopharmaceutical factors that can influence the qualities and performance of the finished product must be taken into account. The researchers have studied all these factors over time and found that the efficiency of entrapping, the morphology of the microspheres and the release of the active substance from the polymeric matrix can be influenced by temperature, the solvent used, the concentrations of polymers and the auxiliary substances used in the manufacturing process. Additionally, the choice of the preparation method depends on the solubility mode of the medicinal substance, the characteristics of the microencapsulation material and the purpose of using the pharmaceutical form [95].

## 4. Factors That Can Influence the Process of Microparticles Formation by Complex Coacervation

### 4.1. Polymer Concentration, Nature and Properties

Polymer concentration is considered the most important factor to be taken into account when developing microspheres because it can affect both the morphology and the dimensions and efficiency of their entrapping. Many studies prove that with the increase in the concentration of the polymer, the efficiency of entrapping of the drug, the average size of the microspheres and the viscosity of the polymeric solution increase [12]. In the case of using a low concentration of polymer, the resulting microspheres will have a low density, large distribution area and a rapid release of the medicinal substance [96].

The nature and properties of the polymer used in the development of microspheres is very important to know when it is desired to develop biocompatible and biodegradable microspheres. Polymers of natural origin are the ideal candidates for obtaining microspheres. By their functions, these polymers are directly responsible for the formation of the microsphere matrix, the way of lining the medicinal substance, the mode of release of the pharmaceutical form and the bioavailability of the active substance [12,97]. When using natural polymers, they can influence the formation, size and surface charge of the microspheres by their molecular weight and degree of deacetylation [50,91].

In complex coacervation, pH adjustment is very important because the complexing can be reached at that pH value in which both polymers are charged in the opposite way. The binding of chitosan macromolecules at critical pH to the chains of alginate first leads to the formation of complexes with low stability, and gradually to insoluble aggregates with increased stability. Until the maximum pH at which the polymers reach the electrical equivalence is reached, macroscopic changes in the turbidity occur in the system [98]. The optimal pH range for reaching a high degree of coacervation differs depending on the nature of the polymers used in the preparation [54]. 

### 4.2. Medicinal Substance

Medicinal substances can influence the efficiency of entrapping and release from the pharmaceutical form by their degree of solubility and acidity constant [34,50,53,91]. Studies show that if the concentration of the medicinal substance is increased, the effectiveness of entrapping also increases, but up to a certain limit, since too much increase can result in a decrease in the efficiency of entrapping. The researchers explained that this phenomenon can occur when a too-high concentration of the medicinal substance is used, i.e., above the load limit of the microencapsulated pharmaceutical form, because then the surplus medicinal substance will migrate from the polymeric matrix to the aqueous dispersion, which is poor in the colloid [12].

### 4.3. Stirring Speed

The stirring speed is an important factor that must be taken into account at the stage of development of the microspheres because the distribution and dimensions of the microspheres depend on it. Studies show that if a high stirring speed is applied in the system, smaller and finer microspheres are produced. Other studies show that with the increase in the speed of stirring, the efficiency of capturing the drug decreases, which is a disadvantage [12].

### 4.4. Stirring Time

The stirring time can influence the entrapping and size of the microparticles as follows: when the stirring time increases, the size of the microparticles increases, but the efficiency of the entrapping of the active substance decreases because with the increase in stirring time, the gelling time also increases, and in the microspheres, they diffuse more calcium ions moving the active substance from there, thus decreasing the entrapping. On the other hand, if resorting to a decrease in the time of reticulation, then incomplete gelling occurs in the system, which results in a decrease in the entrapping of the active substance. In other words, the increase or decrease in the efficiency of entrapping depends primarily on the type of encapsulated active substance as well as on the type of preparation method chosen [12].

### 4.5. Release Time

The release time of the medicinal substance from the matrix is another important factor that depends on the properties of the polymeric matrix and the way of encapsulation of MS in the matrix. In the resulting system, the polymeric matrix controls the release of MS. There are two mechanisms by which the drug can be ceded: by degradation of the polymeric matrix or by diffusion of the medicinal substance through the polymeric network. In the case of microspheres consisting only of biodegradable natural polymers, the cession of the medicinal substance from the polymeric matrix is made both by diffusion and erosion. When the microspheres are exposed to the dissolving medium, the release of MS from the matrix is determined by the diffusion process that causes the swell of the matrix and then the dissolution and erosion of the microspheres [12].

### 4.6. Release Capacity of the Medicinal Product

Knowledge of the kinetics of releasing active compounds from the pharmaceutical form is essential for the effective use of the drug delivery system [99].

The release of active compounds from the alginate matrix of microencapsulated delivery systems can be influenced by: the concentration, physical and chemical properties of the active compound (a high amount of active compounds reduce the diameter of the matrix and the release capacity of the drug), the concentration of alginate, the interaction of alginate and the active compound, the properties of the alginate matrix and the type of encapsulation (in microcapsules, since the polymeric membrane is permeable, they can control the release of the drug from the pharmaceutical form through the degradation mechanism of the alginate network and the diffusion of the active compound through the alginate network, and in polymeric micromatrix, the mechanism by which the release of the active compound from the polymeric matrix occurs is based on the combination of diffusion and degradation), porosity and roughness of the microparticle surface, chemical composition, degradation capacity, size of microparticles and amount of pharmaceutical dosage form [12,99].

Alginate used to obtain controlled-release pharmaceutical systems such as microparticles can favor the release of the microencapsulated medicinal substance by three processes:

The degradation process of the polymeric network (in the case of water-soluble substances);

The process of diffusion through the polymeric matrix as a result of swelling (in the case of substances insoluble in water);

The process of releasing the active compounds from the surface of the microparticles leading to an explosion effect [2,27,99].

The pore size of the alginate matrix is considered to be between 5–200 μm but can be reduced if various factors are taken into account in the preparation, such as the drying process, the technique of preparing the pharmaceutical form and the concentration of sodium alginate used. Thus, it was found that the microspheres should not be subjected to complete dehydration when drying is performed in order to not cause cracking and erosion of the microsphere matrix at rehydration and also to not use too high alginate concentration in the preparation of the pharmaceutical form because the higher the concentration and the porosity is, the more the increase in the water absorption and release capacity of the drug is favored [2,27].

Depending on the nature of the uronic acid groups in the structure, sodium alginate that is richer in G-groups forms a gelatinous matrix with a lighter pore structure and with higher diffusion rates of the microencapsulated material, maintaining integrity for long periods of time. Sodium alginate containing a higher amount in M-groups forms a softer, less porous gelatinous matrix that tends to disintegrate easily over time [2]. The porosity of the gel can also be influenced by the concentration in which the divalent cations were used in the preparation of the gel. When the content of Ca^2+^ ions is higher, the porosity of the matrix and the rate of release of medicinal substances from the pharmaceutical form increases. However, in the case of the use of very high concentrations of Ca^2+^ ions, the release of the drug from the pharmaceutical form is limited by the effect of ionic strength. The Na ^+^ ions in the alginate structure should not be present in large quantities either, as they can compete with gelled Ca^2+^ ions, being able to slow down the gelling process [2].

**Figure 5:**
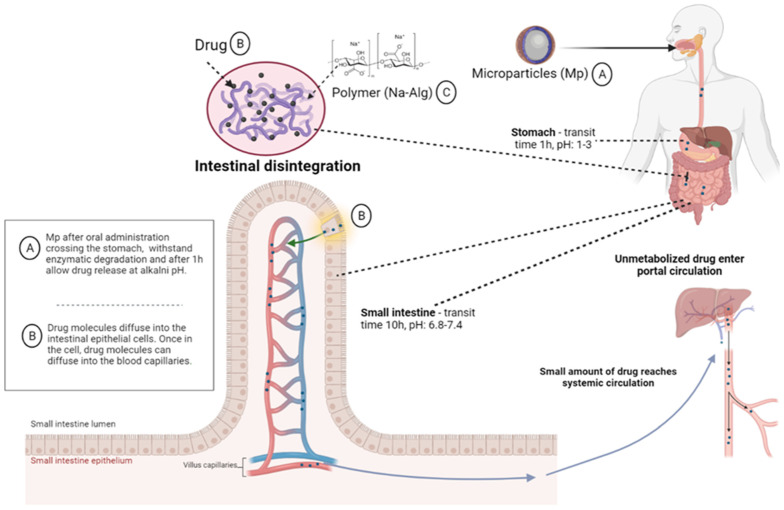
The path traveled by polymeric microparticles from administration to metabolism [102,103] (scheme made with the Biorender program). Legend: Na-Alg—sodium alginate, Mp—microparticles.

The oral route of administration of medicines has some advantages compared to other routes of administration: it is noninvasive, has a very large absorption surface and offers the possibility of entering in the systemic circulation of ingested particles/drugs [100]. After oral administration, the microencapsulated pharmaceutical forms are not affected by the harsh physiological environment of the gastrointestinal tract, since the polymers chosen at the microencapsulation have the ability to protect the active compounds from gastric acidity and enzymatic activity, increasing the solubility in the lumen and the transport of medicinal substances through the gastrointestinal barrier, as can be seen in Figure 5, unlike the classical pharmaceutical forms to which it can cause degradation and loss of action potential. In the small intestine, after the microparticles have crossed the mucus layer and reached enterocytes, part of the medicinal substances will be taken up by the systemic circulation through the portal vein after undergoing a first hepatic passage, and the other part reaches the vena cava through the intestinal lymphatic vessels, where it will manifest its therapeutic effect [100,101].

## 5. The Use of Alginate as a Microencapsulation Material

The category of the newest pharmaceutical forms introduced in therapeutics includes microparticles that have dimensions between 1 and 1000 mm compared to classical oral pharmaceutical forms (powders, capsules, tablets, pills, tablets) as mentioned in the Pharmaceutical Technology Treaty [53].

In the literature for these pharmaceutical forms, several names are used: microparticles, microspheres, microcapsules, micromatrix and micropeletts. According to the Encyclopedia of Pharmaceutical Technology, volume 6, the term “microcapsules” refers to a solid pharmaceutical form consisting of one or more active substances (in the solid or liquid state) which is surrounded by a separate wall of the capsule, consisting of polymers; the term “micromatrix” refers to the polymeric matrix in which the encapsulated active substances are homogeneously dispersed; and the terms “microparticles” and “microspheres” are synonymous, used as general terms. In the Pharmaceutical Technology Treaty, volume 3 [53], only the term “microparticles” is used as a general term. In other studies, other authors use the terms “micromatrix” and “microspheres” or the terms “microcapsules” and “microspheres” as synonyms, as mentioned in the Encyclopedia of Pharmaceutical Technology: Volume 6 [104]. 

According to the Pharmaceutical Technology Treaty and other studies, microencapsulated pharmaceutical forms can be divided into two categories of microparticles: microcapsules in which the active substance is surrounded by a protective polymeric coating, and microspheres in which the active substance is dispersed or dissolved in the polymer matrix [53]. Dhamecha, D. et al., have revealed that microencapsulation is the technology through which small, bioavailable microencapsulated delivery systems can be developed, such as microspheres or microcapsules that can protect active substances from the unfavorable physiological environment during their passage through the highly acidic environment of the stomach, from external factors (light, humidity, heat, air) or from enzymatic degradation, while also ensuring the release of the drug in a certain place in the body [18,105]. 

Several studies have shown that various microencapsulation techniques are currently available: the air suspension method, spray-drying, coacervation, extrusion, vibrational jet, spinning disk, supercritical fluid precipitation, freeze-drying, emulsification/gelation method, etc., and the resulting microencapsulated products are widely used in the pharmaceutical, biomedical, agricultural, food and cosmetic industries [53,104,106,107,108,109,110].

**Figure 6:**
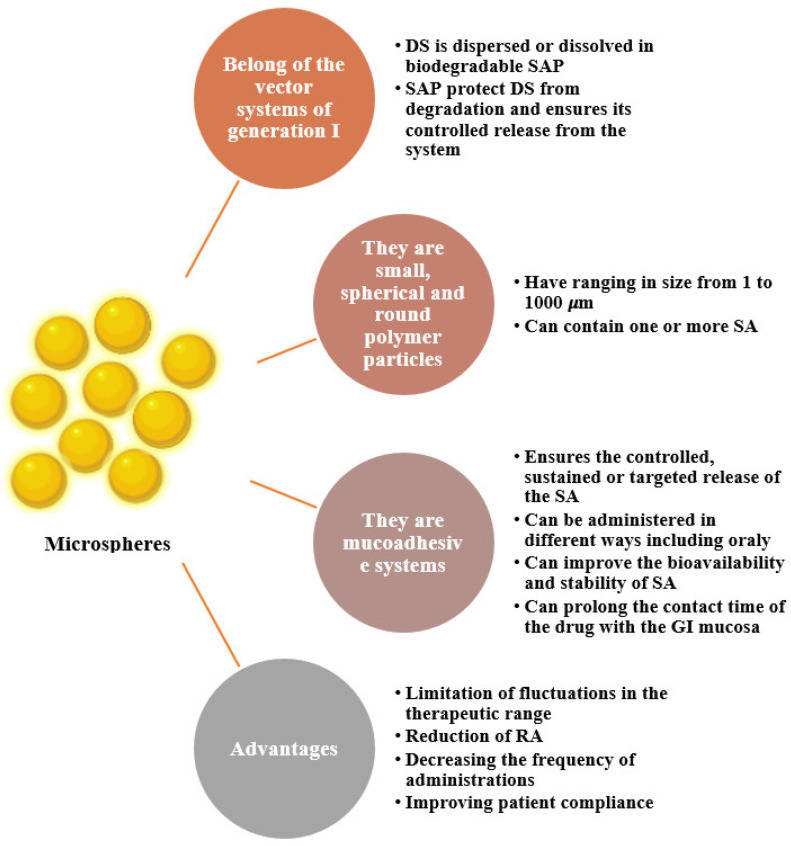
Characterization of microsphere-type microparticles [34,53,59,111,112,113,114,115]. Legend: DS—drug substance, SAP—polymeric auxiliary substance, SA—active substance, GI—gastrointestinal, RA—adverse reactions.

In Figure 6, we present a brief characterization of the microencapsulated pharmaceutical forms.

**Figure 7:**
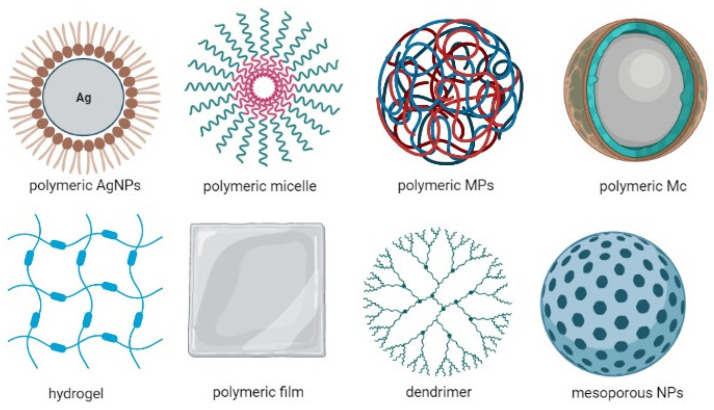
Structural representation of some of the most representative pharmaceutical forms in which sodium alginate is used (scheme made with the Biorender program). Legend: AgNPs—silver nanoparticles, MPs—microparticles, Mc—microcapsules.

In the last 25 years, research has focused on the development of biodegradable polymeric microspheres (Freiberg, S., et al.) because the encapsulation of medicinal substances (Figure 7) in such systems has proven to be advantageous: microspheres can be administered in different ways (oral, parenteral, cutaneous, etc.); depending on the mode of preparation, they can provide desired release profiles of medicinal substances, and in some cases, they can direct the release of medicinal substances to a particular organ, mask the unpleasant taste of some pharmacologically active compounds, reduce gastrointestinal irritation, decrease adverse effects, etc. [97,106].

Biocompatible and biodegradable polymers are increasingly being studied in the biomedical and pharmaceutical fields because they have a chemical structure similar to that of macromolecules in the native extracellular environment, and unlike synthetic polymers, are compatible with living cells [7]. From the category of natural anionic polymers, sodium alginate is the most used in pharmaceutical applications, as mentioned in work performed by Varma, K., et al. [116].

Alginates in the pharmaceutical field can be used as disintegrants and binders of tablets, viscosity-modifying agents, stabilizers of the dispersal system, in the production of suspensions and emulsions, and as thickeners. Being a natural polymer, it can be used to obtain hydrogels (Augst, A.D., et al.), polymer films (Ning, H., et al.) and polymeric micelles (Manna, K., et al.). It can be included in the matrix of micro-/nanocapsulated pharmaceutical forms with controlled release of the type of microspheres, microcapsules, silver polymeric nanoparticles (Gudimalla, A., et al.), silicon mesoporous polymeric nanoparticles or when obtaining anticancer dendrimers (Liu, C., et al.), etc., because it is biodegraded by the body without producing toxic effects, is biocompatible, can form gels and hydrogels easily, is inert and chemically compatible with many natural/synthetic materials, can be easily synthesized and manipulated and can control the speed of release of active compounds from pharmaceutical systems [4,105,117,118,119,120,121,122,123,124].

Table 2 highlights some examples of how to obtain microencapsulated pharmaceutical forms based on sodium alginate and their advantages. Studies show that sodium alginate used in the microencapsulation of various active substances, when used in conjunction with other polymers, proteins or enzymes, leads to microencapsulated pharmaceutical forms with increased stability and bioavailability [7]. In the microspheres with alginate, different types of medicinal substances of natural or synthetic origin can be caught, including even cells, bacteria, proteins, peptides or nucleic acids, as shown by the study of Dhamecha, D., et al. [105].

Microparticles made up of mucoadhesive polymers can improve the permeability of encapsulated medicinal substances of a protein nature (insulin) through the intestinal mucosa [125]. Thus, alginate microspheres can be used as pharmaceutical systems for oral insulin administration [126] because alginate slowly degrades in the body, protects the active substance from enzymatic degradation, improves its penetration through the intestinal wall [113] and facilitates the systemic absorption of insulin. Together with chitosan, it has been successfully used in the development of dietary supplements based on mangosteen extract, with an antioxidant effect [127]. Yang D. et al., obtained microparticles of <220 μm size, with high rates of release of amoxicillin from the pharmaceutical form due to the large specific surface area. Due to the distribution of chitosan to the surface of the alginate microspheres, the authors claim that the release time of MS is prolonged, and the stability of the microspheres is increased [128].

**Figure 8:**
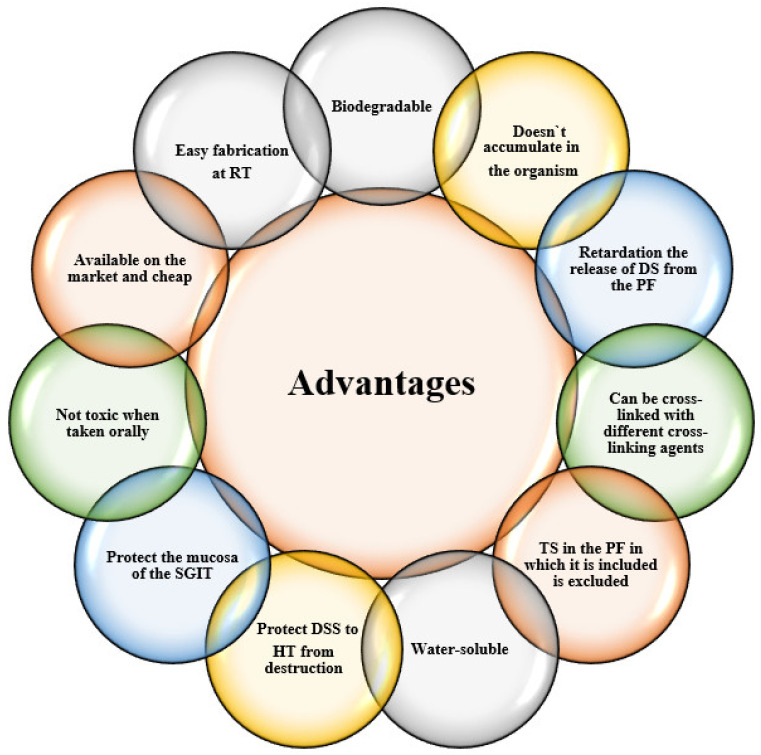
Advantages of using alginate in the development of pharmaceutical forms with controlled drug release [4,153,154,155]. Legend: DS—drug substance, DSS—drug sensitive substance, RT—room temperature, HT—high temperature, TS—toxic solvents, PF—pharmaceutical form, SGIT—superior gastrointestinal tract.

Other studies show that alginate can also be used in the development of antituberculosis nanoparticles that can be inhaled directly into the lungs. The nanoparticles obtained showed increased bioavailability, low toxicity and sustained drug release for long periods of time [17]. Samani S.M. et al., have developed a multiparticulate and mucoadhesive system intended for the administration of nystatin on the oral mucosa, which is much more advantageous in treating pathologies of the oral mucosa than classical forms of administration. This pharmaceutical form is actually a carbopol-based mucoadesive gel in which nystatin is encapsulated in alginate microparticles capable of masking its unpleasant taste, ensuring the controlled release of the active substance from the microspheres and prolonging its contact time with the oral mucosa [129]. Also, main advantages of alginate usage into the development of pharmaceutical forms are presented in Figure 8.

**Table 2:** Potential uses of sodium alginate microparticles.

Preparation Technique	Advantages	Active Substance	Potential Apps	Reference
Emulsification with pectin, Na caseinate and whey protein	Development of effective MPs, with a diameter between 45–70 μm, with high swelling and release rates of extract	Olive leaf extract	MPs	[130]
Coacervation with mucina	Oral administration of microencapsulated and enteric-coated insulin can control blood sugar effectively	Insulin	MPs	[131]
Multiple U/A/U emulsification using sunflower oil and Span 80	Good antioxidant and antimicrobial properties, and in vitro studies have shown an initial release in the form of an explosion followed by slow release	Essential oil of *Satureja hortensis*	MPs	[132]
Emulsification with HPMC using tween 85 and CaCl_2_ dihydrate	Administration of chemotherapeutic agents by inhalation route, directly into the lungs, in the therapy of cancer	Paclitaxel	MPs	[133]
Spray-drying and ionotropic gelling with Ch and CaCI_2_	Potential administration to the colon for the treatment of IBD	5-aminosalicylic acid	MPs	[134]
Extruders with denatured whey protein	Carrier of promising drugs to improve oral administration of insulin	Insulin	MPs	[135]
Emulsification with paraffin oil and tween 80	Simple and economical encapsulation method that allowed the controlled release of the drug from FF	Diclofenac sodium	MPs	[136]
Coacervation with Ch	Alternative to treat tuberculosis	Rifampicin	MPs	[137]
Microfluidic method with gelatin and CaCI_2_ dihydrate	Ensures the intestinal release of MS	Ketoprofen	MPs	[138]
Extruders with CaCI_2_ and Ch	Directing the action of MS in the lower parts of the GI tract and EE >75%	Naproxen	MPs	[139]
Spray-drying with Ch and CaCI_2_ and enteric coating with Eudragit S100	Local treatment of IBD	Budesonide	MPs	[140]
Reticulation with CaCI_2_ and Ch by spray-drying	Increased BD MS at the tumor site for a longer period of time and provides a specific release into the lymphatic system	Tamoxifen	MPs	[141]
Spray-drying with Ch	According to in vitro release studies complexation with Ch controlled the release of MS from MPs and increased their BD	Metoclopramide	MPs	[142]
Method of ionic gelling of Ch with Na-TPP and coverage with Na-Alg	Covering Ch-MPs with a layer of Na-Alg increases Ms’s resistance to gastric degradation and prolongs the release of MS from FF at the intestinal level	Metoprolol succinate	MPs	[143]
Spray-drying with CaCI_2_	Spray-drying can achieve Mucoadhesive Ms with high EE and high production yield	Metformin	MPs	[144]
The emulsification/external gelation method with CaCI_2_, isopropanol, tween 80, paraffin oil and bis-(1,3-dibutylbarbituric acid) trimethine oxonol	The development of these FF has significantly reduced some of the adverse effects of amphotericin B	Amphotericin B	MPs	[145]
The coacervation technique with Ch	It allows obtaining controlled-release MPs, which have a rough surface from which MS are released through the diffusion process	Vancomycin chloride	MPs	[99]
The emulsion-cross-linking method with liquid paraffin, Span 80, methanol, sopropyl alcohol and CaCI2 as cross-linker	Obtaining enteric release Ms due to MS release in alkaline pH medium, with high entrapment EE 91% of bioactive hydrophilic compounds	Isoniazid	Ms	[146]
The emulsification–cross-linking method cu HPMC folosind hexane, Span 80, CaCI_2_, isopropyl alcohol	Development of FF for intranasal administration of MS	Metoprolol tartrate	Ms	[147]
The extrusion technique using CaCI_2_	Possibility of incorporating probiotics into the microencapsulated FF matrix	Lactobacillus acidophilus.	MPs	[148]
The emulsification method with magnesium stearate using liquid paraffin, Span 80, calcium chloride, isopropyl alcohol	Getting Ms with sustained release	Ibuprofen	Ms	[115]
The spray-drying technique with CaCI_2_	Possibility of proteic MS encapsulation in microparticulate FF for oral administration	Insulin	MPs	[149]
The spray-drying technique with CaCI_2_	The release of FF at the intestinal level where following the process of swelling and then erosion is released MS	Caffeine	MPs	[150]
The ionotropic-gelation technique using polysaccharide extracted from seeds of *Tamarindus indica* L. and CaCl_2_ as cross-linker	Preparation of intestinal-release FF for 10 h with EE of 94.86 ± 3.92% SM	Metformin HCl	MPs	[151]
The aerosolization technique using CaCl_2_ as cross-linker and maltodextrin as lyoprotectant	Obtaining high FF entrapping of the drug due to the high concentration of Na-Alg used, of spherical shape and smooth surface due to the use of maltodextrin	Metformin HCI	Ms	[152]

Legend: Na—sodium, U/A/U—oil/water/oil, CaCl_2_—calcium chloride, Ch—chitosan, Na-TPP—sodium tripolyphosphate, IBD—inflammatory bowel disease, MPs—microparticles, FF—pharmaceutical forms, SM—medicinal substance, GI—gastrointestinal, EE—entrapment efficiency, BD—bioavailability, Ch-Mps—chitosan microparticles, Na-Alg—sodium alginate, Ms—microspheres.

## 6. Conclusions

In conclusion, alginate can be used for the development of various pharmaceutical forms, including the obtaining of microparticles, because the formation of gelatinous matrix/coating is carried out under gentle conditions, at room temperature and using reagents/biocompatible materials if a preparation technique such as complex coacervation with another polymer including chitosan is used. In the case of its use in obtaining microparticles, the release of drugs from the alginate microspheres is carried out mainly through the process of diffusion through the pores of the polymeric matrix at a certain pH value due to its erosion, as shown by Sachan, N.K. et al. and Simó, G. et al. [4,20].

The macromolecules of natural origin have attracted the attention of many researchers as being essential to protect the structures of unstable drug substances. After analyzing the studies carried out by various authors, we found that these molecules are used for both investigational and therapeutic purposes. This requires the design of certain drug delivery formulations knowing the nature of the macromolecule, its target organ, the required dose and the route of delivery. Therefore, we consider it important to use sodium alginate to optimize the delivery of drug substances for maximum therapeutic performance in the body after administration.
